# Human herpesvirus-6 infection in a critically ill and immunocompetent patient: a case report

**DOI:** 10.1186/s13256-024-04387-5

**Published:** 2024-03-01

**Authors:** Xin Tian Chia, Hai Liang Marc Wong, Jia Shen Loh

**Affiliations:** 1 Ministry of Health Holdings, Singapore, Singapore; 2 Department of Internal Medicine, Sengkang General Hospital, Singapore, Singapore; 3 Infectious Disease, Farrer Park Hospital, Singapore, Singapore

**Keywords:** Human herpesvirus-6, Immunocompetent, Chromosomally integrated HHV-6

## Abstract

**Background:**

Human herpesvirus-6 is a rare infection in an immunocompetent adult. In existing literature, there is a dearth of knowledge that mainly exists as case reports and case series.

**Case Presentation:**

In this case report, we described a 29-year-old female of Myanmarese descent patient from Myanmar who presented with altered mental status and non-specific respiratory and gastrointestinal symptoms. She was initially treated for pneumonia and discharged well. However, she re-presented to the hospital and was subsequently treated for severe central nervous system infection. Cerebrospinal fluid studies detected human herpesvirus-6 polymerase chain reaction with associated high serum human herpesvirus-6 concentration. This infection also triggered hemophagocytic lymphohistiocytosis. Treatment was initiated against both human herpesvirus-6 infection and hemophagocytic lymphohistiocytosis, and she responded to antiviral treatment and steroids, respectively.

**Conclusion:**

This case study highlights the need for prompt diagnosis and treatment of this severe disease and the dangerous complications. Additionally, the authors share insights on the diagnostic challenges faced in the treatment of this patient.

## Background

Human herpesvirus-6 (HHV-6) is a member of the *herpesviridae* family that is thought to be a ubiquitous infection that approaches 100% seroprevalence in the human population [1]. HHV-6 also belongs to the subfamily *betaherpesvirinaie*, which is characterized by an established latency in leukocytes after the initial infection [2]. Furthermore, HHV-6 also demonstrates neurotropism for glioblastoma and neuroblastoma cell lines [2]. It commonly causes primary childhood infections known as roseola infantum (sixth disease), which is a self-limiting disease. Although rarely associated with serious or fatal complications, it can lead to disseminated or neurological manifestations including seizures, meningitis, fulminant hepatitis [1]. Re-activation of HHV-6 infection in the adult population is typically reported among immunocompromised individuals, such as recipients of organ or hemotopoetic stem cell transplants or individuals who have human immunodeficiency virus (HIV). Similarly, such re-activations can lead to severe hepatitis, pneumonitis, and even lethal meningoencephalitis [3, 4]. On the contrary, re-activation of HHV-6 in an immunocompetent host is rare and exists mainly in literature as case reports [5]. Herein, we report a case of possible HHV-6 infection precipitated by severe illness complicated by multiorgan failure in an immunocompetent patient.

## Case presentation

Our patient is a 29-year-old female of Myanmarese descent from Myanmar who works as a domestic worker with minimal access to healthcare systems. She has no prior medical history or significant family history. She first presented to hospital with 2 weeks of intermittent fever that was associated with cough, vomiting, and diarrhea. In the emergency department (ED), the patient was febrile at 40 °C and hypotensive with a blood pressure of 80/61 mmHg. Physical examination showed that patient was drowsy and disoriented. Neurological examination was significant for bilateral reduced abduction suggestive of bilateral sixth nerve palsy, which showed interval improvements on repeated assessments. Lungs were clear on auscultation and abdominal examination was unremarkable without focal tenderness or guarding on palpation.

Initial investigations showed elevated inflammatory markers with a white blood cell (WBC) count of 31 × 10^9^/L, procalcitonin (procal) of 193.6 µg/L, and C-reactive peptide (CRP) of 218.2 mg/L, which were suggestive of an infective process. Computed tomography (CT) imaging of the brain, which was performed on admission to evaluate for structural etiologies of the altered mental status, was reported normal.

In view of severe shock secondary to presumed infection, she was treated with fluid resuscitation in the ED and required single inotropic support in a high-dependency unit for 1 day. The admitting team also initiated her on intravenous (IV) ceftriaxone, vancomycin, and acyclovir to treat presumptively for meningoencephalitis in view of the initial presentation of altered mental status with sepsis. As the source of sepsis remains unclear, CT abdomen and pelvis was subsequently performed. The CT scan demonstrated only patchy consolidative and ground-glass opacities in the lung bases without any focal intra-abdominal collection. Guided by the CT scan findings, IV ceftriaxone, vancomycin, and acyclovir were stopped, and she was treated for severe pneumonia with IV ceftazidime and levofloxacin instead. However, her recovery was slower than expected, which led to a CT thorax to evaluate the lungs for complicated infections, such as abscesses. Following which, findings from this CT thorax scan were consistent with the diagnosis of pneumonia without any evidence of abscesses or infective collections.

The patient was observed to improve clinically with improvements in hemodynamics stability, respiratory status, and mentation. Hence, further investigations including cerebrospinal fluid (CSF) studies were deferred. The patient completed 7 days of IV ceftazidime and levofloxacin during admission and was planned to complete antibiotics treatment with another 14 days of oral levofloxacin. On discharge, the patient was stable, and inflammatory markers improved to WBC of 6.22 × 10^9^/L, procal of 121.60 µg/L, and CRP of 14.8 mg/L, respectively. No definite infective etiology was identified upon discharge.

However, she re-presented to the ED 8 days later with similar symptoms of fever, cough, abdominal pain, nausea, and vomiting. Objectively, the patient was febrile with 38.8 °C and in extremis with severe hypotension with a blood pressure of 77/42 mmHg, and severe hypoxia with a saturation of 65% on room air. The patient was also drowsy with generalized rash on examination. The rest of the examination was unyielding with no signs of acute abdomen. She was intubated emergently in the ED for airway protection and was admitted to the intensive care unit (ICU) on dual inotropic support.

Initial investigations were suggestive of severe septic shock with elevated inflammatory markers (CRP of 86.1 mg/L and procal of 62.2 µg/L) and lactic acidosis (lactate 4.4 mmol/L). The initial septic screen showed increased opacities on the chest X-ray, and she was treated with a working diagnosis of severe pneumonia. Given the severity of illness, infectious disease consult was also promptly obtained. Consequently, the infectious disease team then advised to consider meningoencephalitis as a differential, as lung infection may not sufficiently account for the severity of septicemia and the patient’s altered mental status. However, the medical team was unable to proceed with an immediate lumbar puncture, as the patient had severe coagulopathy from disseminated intravascular coagulation (DIVC) on admission. Coagulation profile was deranged with a prothrombin time (PT) of 16 seconds and an activated partial thromboplastin time (aPTT) of 118.4 seconds. A lumbar puncture was subsequently performed on Day 4 of admission when the coagulopathy was corrected, and the patient was clinically stable. There was a high opening pressure of 41 cm CSF, and CSF studies were sent for a broad virology and microbiology screen, which included a multiplex assay. Significantly, the meningitis multiplex assay (Biofire FilmArray) detected a positive HHV-6 polymerase chain reaction (PCR). This corresponded with findings of high HHV-6 serum (cell-free) concentration of 4,805,000 (6.68 log) copies/mL. CSF bacterial cultures and the rest of the viral screen returned negative. Blood cultures taken on Day 1 of the prevailing admission reported *Brevibacterium* species, which was deemed to be a contaminant as it was not isolated in subsequent blood cultures. Significant biochemical and microbiological investigations are summarized in Tables 1 and 2.

**Table 1:** Significan﻿t investigations done throughout admission

Biochemistry	Days of admission							
	1	2	5	7	12	18	21	28
Procalcitonin (µg/L)	62.2	28.6	94.2	14.4	0.88	NA	NA	NA
CRP (mg/L)	86.1	56.6	13.5	10.8	7.1	NA	11.4	NA
Ferritin (µg/L)	NA	98,870	NA	7410	NA	NA	590	NA
Hematology								
Hemoglobin (d/L)	9.9	9.9	9.0	7.1	9.1	10.9	10.1	10.9
WBC count (× 10^9^/L)	1.01	1.02	23.51	22.38	9.02	5.67	8.21	8.44
Neutrophil (× 10^9^/L)	0.56	0.40	25.50	15.33	8.28	4.09	6.99	5.93
Lymphocyte (× 10^9^/L)	0.26	0.52	18.95	1.52	0.49	0.84	0.81	1.98
Platelets (× 10^9^/L)	60	52	34	29	272	232	146	223
Coagulation								
PT (s)	NA	16.5	11.9	11.4	NA	11.3	11.1	10.6
aPTT (s)	NA	118.4	37.3	27.3	26.6	30.9	33.5	29.0
INR	NA	1.64	1.12	1.09	NA	1.08	1.06	1.01
Virology								
*Cytomegalovirus* (CMV, IU/mL)	NA	120 (2.08 log)	NA	NA	NA	NA	NA	NA
HHV-6 PCR (copies/mL)	NA	NA	4,805,000 (6.68 log)	NA	63,425 (4.80 log)	43,000 (4.63 log)	96,825 (4.99 log)	42,400 (4.63 log)

Not applicable (NA) indicates that the investigation was not repeated on the day; *CRP* C-reactive Peptide, *WBC* White Blood Cell, *PT* Prothrombin Time, *aPTT* Activated Partial Thromboplastin Time, *INR* International Normalised Ratio, *HHV-6 PCR* Human Herpesvirus-6 Polymerase Chain Reaction

**Table 2:** Significant cerebrospinal fluid studies

**Biochemistry**	
Glucose (mmol/L)	5.61 (serum glucose 10.1)
Protein total (g/L)	0.54
**Cell differentials**	
RBC (/µL)	1091
WBC (/µL)	23
Lymphocytes (/µL)	11
Polymorphs (/µL)	70
**Microbiology**	
Aerobic culture	No bacterial growth
**Meningitis multiplex assay**	
*Escherichia coli* K1	Not detected
*Haemophilus influenza*	Not detected
*Listeria monocytogen*	Not detected
*Neiss meningitidis*	Not detected
*Strep agalactiae*	Not detected
*Strep pneumoniae*	Not detected
*Cytomegalovirus*	Not detected
*Enterovirus*	Not detected
Herpes simplex V1	Not detected
Herpes simplex V2	Not detected
Human herpes virus 6	**Detected**
Human *parechovirus*	Not detected
Varicella zoster virus	Not detected
*Cryptococcus neoformans*/*gattii*	Not detected

*RBC* Red Blood Cell, *WBC* White Blood Cell

As part of the evaluation for HHV-6 encephalitis, an immunodeficiency screen was duly performed. However, the patient was a domestic worker in a foreign land with financial constraints. This inevitably limited the workup of possible immunodeficiencies, which was another diagnostic challenge encountered by medical team. Hence, the patient was only screened for basic immunodeficiencies, which demonstrated that she was HIV negative. CD4 counts was measured on Day 7 of admission and was likely confounded by the ongoing sepsis. Hence, the low CD4 cell counts (CD4 absolute: 416 and T-cell CD4: 30.7%) was difficult to interpret and, likely an inaccurate representation of the immunity status. She was also screened for autoimmune diseases with investigations such as antinuclear antibody, double-stranded deoxyribonucleic acid (dsDNA) antibody, C3, C4 and anticardiolipin antibodies, which all returned negative.

For treatment, she was first initiated on empirical antibiotics coverage with 500 mg IV azithromycin every 24 hours, 1250 mg vancomycin every 12 hours, and 2 g meropenem every 8 hours to presumptively treat for bacterial meningitis and recurrent pneumonia. Treatment was then changed to 200 mg IV ganciclovir every 8 hours on Day 4 of admission once CSF results were suggestive of HHV-6 encephalitis. The treatment of this illness was also complicated by multisystemic involvement. Firstly, the patient also had severe acute respiratory distress syndrome (ARDS) and required a period of prone ventilation and intubation in the ICU. Secondly, investigations demonstrated an elevated ferritin of 98,870 µg/L, triglycerides of 8.49 mmol/L, d-dimer > 35.20 mg/L FEU, fibrinogen of 1.01 g/L, and aspartate aminotransferase (AST) of 645 U/L, coupled with pancytopenia [hemoglobin (Hb) of 9.9 d/L, WBC of 1.01 × 10^9^/L, and platelet count of 60 × 10^9^/L], which were suggestive of secondary hemophagocytic lymphohistiocytosis (HLH). The H-score at the time of diagnosis was 250, and hematology service was consulted for management of HLH [6]. Bone marrow aspirate was not performed in view of the relative certainty of diagnosis of HLH. She was treated with IV steroids and etoposide was deemed not necessary since she was also on treatment for HHV-6 infection, which was the presumed precipitant of HLH.

After the initiation of ganciclovir and steroids, the patient demonstrated good clinical response. In the ICU, she was weaned off inotropic support by Day 3 of admission and was successfully extubated on Day 10 of admission. Follow-up laboratory findings showed steady downward trend of HHV-6 serum titers from 63,425 (4.80 log) copies/mL on Day 11 of admission to 42,400 (4.63 log) copies/mL on discharge. Likewise, there was gradual resolution of coagulopathy with normalized coagulation profile (aPTT 27.3 seconds and PT 10.7 seconds), with improvement in blood cell counts (Hb of 10.0 d/L, TW of 13.61 × 10^9^/L, and platelet count of 359 × 10^9^/L) on discharge.

She eventually completed a total of 21 days of intravenous antiviral therapy with IV ganciclovir and was continued on another 33 days of 450 mg oral valganciclovir once daily. For HLH treatment, she completed 14 days of IV steroids, followed by another 44 days of weaning doses of oral steroids with trimethoprim/sulfamethoxazole prophylaxis. Prior to discharge, the patient required 2 to 3 weeks of intensive rehabilitation to achieve her premorbid functional status. On discharge, she returned to her home country with no further follow-up at our hospital.

## Discussion and conclusions

HHV-6 infection is a rare manifestation in an immunocompetent individual. Besides a handful of case reports, there are no large-scale review papers or controlled trials available in current literature. Hence, we aim to review this case study in association with existing literature and provide a discussion on the diagnostic challenges faced.

A review of existing cases reports did not illustrate any clear predispositions or clinical attributes that differentiate HHV-6 meningoencephalitis from other viral etiologies in immunocompetent hosts. Most of the initial presentation included non-specific altered mental status, seizures, and focal neurological signs [5, 7–9]. CSF studies were generally suggestive of a viral etiology but demonstrated a wide range of CSF WBC from 16 to 535 and CSF protein from 45 to 533 [10]. Neuroimaging was inconsistently performed in these reports, with most reporting abnormal and varied findings, such as ventricular enlargements, hypodense lesions, and temporal lobe abnormalities [5, 7, 10, 11]. Final diagnosis was eventually made with HHV-6 PCR detection in CSF samples [5, 8, 9]. This highlights the importance of early CSF studies in patients with otherwise unexplained mental obtundation to ensure timely diagnosis and treatment.

Similar to studies in literature, the diagnosis of HHV-6 infection was eventually clinched with positive PCR detection in our patient. However, the interpretation of the HHV-6 PCR studies is challenging, as HHV-6 has been found to incorporate into the host genome and persists as chromosomally integrated HHV-6 (ciHHV-6) [12]. For example, one study showed that HHV-6 could be detected in CSF samples from patients who were deemed clinically unlikely to have encephalitis [13]. Furthermore, one HHV-6 study in the UK has demonstrated a prevalence of 0.8% of chromosomally integrated HHV-6 (ciHHV-6), with high HHV-6 viral copies in the serum (> 6 log copies/mL) of healthy immunocompetent individuals [12]. Hence, the ability to differentiate active infection and/or reactivation of HHV-6 from ciHHV-6 is essential in achieving a correct diagnosis.

Literature has reported that both qualitative and quantitative HHV-6 PCR in serum or plasma are not conclusive in differentiating ciHHV-6 from active infection [14, 15]. Instead, it is suggested that PCR analyses should be performed with whole blood or isolated peripheral blood mononuclear cells (PBMCs) [14, 15]. Individuals with ciHHV-6 are expected to have higher levels of HHV-6 titres which are typically > 5.5log10 copies/ml in whole bloods or PBMCs [14]. Additionally, a ratio of 1:1 viral-to-human genome is also highly predictive of ciHHV-6 instead of active infection or reactivation [14].

In our case study, the clinical laboratory performed the analysis of HHV-6 PCR in cell-free serum and we were not able to procure further specimens of whole blood or PBMCs for further analyses. Hence, this will inevitably confound the interpretation of the viral serum titers, which remained high at 42,400 (4.63 log) copies/mL, despite 3 weeks of IV antiviral therapy in our patient. However, the overall clinical improvement observed in our patient remains consistent with treatment response and our clinical assessment of a resolving infection.

In our evaluation of the patient, we faced several challenges with clinching of the diagnosis and monitoring of the treatment progress.

Firstly, we were unable to determine the role of HHV-6 in the initial presentation as there was low clinical suspicion and HHV-6 was not evaluated for  during the first admission. The initial presentation, if due to HHV-6 infection, would be deemed to be self-resolving without any directed treatment of antivirals and steroids.

Secondly, the interpretation of our CSF studies could be confounded by contamination of serum blood from a traumatic lumbar puncture with high red blood cells (RBC) of 1091 detected in the CSF sample (Table 2). Hence, this undermines the diagnosis of HHV-6 encephalitis, as the positive HHV-6 PCR detected in the CSF could be secondary to cross contamination from the serum. Furthermore, quantitative HHV-6 CSF titers are not routinely performed at our center. Nevertheless, the clinical features identified on clinical assessment, such as high opening pressure (41 cm CSF), corresponding initial neurological manifestations of bilateral cranial nerve sixth nerve palsy, and encephalopathic symptoms, were aligned with the diagnosis of encephalitis, which further support our interpretation of the CSF findings.

Lastly, further evaluation of this patient for novel mechanisms of immunodeficiencies was not possible due to financial and social constraints. As our patient required steroids throughout the entire hospital stay, investigations of immune function, which necessitated the cessation of steroids, could not be performed. Regrettably, she also returned to her home country on discharge, and there was no such opportunity to proceed with further investigations or to repeat CD4 counts post-recovery.

In summary, we describe a rare case of HHV-6 infection in an immunocompetent patient with a critical illness. Through this discourse on the interplay of clinical judgement and the challenges faced in the diagnosis of HHV-6 infection, we hope to contribute to the clinical knowledge in the management of this rare disease entity amidst the paucity of data and evidence.

## Data Availability

Not applicable.

## Abbreviations

HHV-6: Human herpesvirus-6
HIV: Human immunodeficiency virus
ED: Emergency department
WBC: White blood cell
Procal: Procalcitonin
CRP: C-reactive peptide
IV: Intravenous
CT: Computed tomography
CSF: Cerebrospinal fluid
ICU: Intensive care unit
DIVC: Disseminated intravascular coagulation
PT: Prothrombin time
aPTT: Activated partial thromboplastin time
INR: International normalized ratio
ARDS: Acute respiratory distress syndrome
Hb: Hemoglobin
dsDNA: Double-stranded deoxyribonucleic acid
ciHHV-6: Chromosomally integrated HHV-6
PCR: Polymerase chain reaction
PBMCs: Peripheral blood mononuclear cells
RBC: Red blood cells
EBV: Epstein–Barr virus
CMV: *Cytomegalovirus*
STAT: Signal transducer and activator of transcription
